# Mobile Phone Apps for Smoking Cessation: Quality and Usability Among Smokers With Psychosis

**DOI:** 10.2196/humanfactors.5933

**Published:** 2017-03-03

**Authors:** Joelle C Ferron, Mary F Brunette, Pamela Geiger, Lisa A Marsch, Anna M Adachi-Mejia, Stephen J Bartels

**Affiliations:** ^1^ Health Promotion Research Center at Dartmouth Department of Psychiatry Dartmouth Hitchcock Medical Center Concord, NH United States; ^2^ Center for Technology and Behavioral Health Department of Psychiatry Dartmouth College Lebanon, NH United States; ^3^ Health Promotion Research Center at Dartmouth The Dartmouth Institute Dartmouth College Lebanon, NH United States

**Keywords:** mHealth, mobile apps, smoking cessation, schizophrenia, psychotic disorders

## Abstract

**Background:**

Smoking is one of the top preventable causes of mortality in people with psychotic disorders such as schizophrenia. Cessation treatment improves abstinence outcomes, but access is a barrier. Mobile phone apps are one way to increase access to cessation treatment; however, whether they are usable by people with psychotic disorders, who often have special learning needs, is not known.

**Objective:**

Researchers reviewed 100 randomly selected apps for smoking cessation to rate them based on US guidelines for nicotine addiction treatment and to categorize them based on app functions. We aimed to test the usability and usefulness of the top-rated apps in 21 smokers with psychotic disorders.

**Methods:**

We identified 766 smoking cessation apps and randomly selected 100 for review. Two independent reviewers rated each app with the Adherence Index to US Clinical Practice Guideline for Treating Tobacco Use and Dependence. Then, smokers with psychotic disorders evaluated the top 9 apps within a usability testing protocol. We analyzed quantitative results using descriptive statistics and *t* tests. Qualitative data were open-coded and analyzed for themes.

**Results:**

Regarding adherence to practice guidelines, most of the randomly sampled smoking cessation apps scored poorly—66% rated lower than 10 out of 100 on the Adherence Index (Mean 11.47, SD 11.8). Regarding usability, three common usability problems emerged: text-dense content, abstract symbols on the homepage, and subtle directions to edit features.

**Conclusions:**

In order for apps to be effective and usable for this population, developers should utilize a balance of text and simple design that facilitate ease of navigation and content comprehension that will help people learn quit smoking skills.

## Introduction

Over half of the people with psychotic disorders such as schizophrenia smoke (45-80%) [1,2]. Evidence-based cessation treatments for tobacco use disorder are effective in people with schizophrenia [3]—combined psychosocial treatment with medication to treat nicotine dependence (nicotine replacement, bupropion, or varenicline)—increase the likelihood of quitting more than twofold over placebo [3]. Unfortunately, most of these treatments are not available to people with psychotic disorders. Strategies to increase access to cessation treatment are needed.

A growing number of treatments developed for smoking cessation are delivered via the Web [4], telephone, and text [5], and more recently through mobile apps [6], increasing access to interventions for smoking cessation. Our research group recently found that typical cessation websites were not usable by people with schizophrenia [7], who have cognitive impairments and less experience with technology. Websites constructed with the Flat Explicit Design Model (FEDM) is most usable by this group [8,9]. This model includes (but is not limited to) a flat design (no more than 2 levels), descriptive labels (vs succinct, without abstract symbols), and text written at a low reading level [10].

When carefully designed, mobile apps may also effectively deliver interventions to clinical populations [8-14]. One app was recently developed and tested for symptom management in people with schizophrenia [13]. In this pilot study, 87% used the mobile app daily for a month, and the majority of participants reported that the mobile app was useful (ie, helped them manage their symptoms) and usable (ie, it was easy to find the information they needed). Whether smoking cessation apps are usable by smokers with psychotic disorders who have cognitive impairments and lower mobile phone experience is largely unknown. One recent study examined the long-term use of a smoking cessation app in 5 adults with severe mental illness and found the usability to be below average [15], indicating that off-the shelf apps may fair poorly for this population.

Although only a few apps have been tested in research studies [15-17], many apps for smoking cessation are publicly available. Abroms et al [18,19] completed two reviews of content and quality of smoking cessation apps: one of iPhone apps in 2009 and one of both Android and iPhone apps from 2012. Both studies found that overall the content of publicly available smoking cessation apps had a very low adherence to clinical practice guidelines [18,19]. The more recent review found that none of the apps connected users to a Quitline, few assisted with a quit plan, and overall recommendations to use medications or to refer to other relevant treatment was poor to nonexistent [18].

Although apps have been assessed for content, we are not aware of any research evaluating many smoking cessation apps for usability among disadvantaged populations who are most likely to smoke and have difficulty accessing cessation interventions. This study begins to fill this gap. In this study, experts evaluated and characterized a large random sample of smoking cessation apps. Then, smokers with schizophrenia and other psychotic disorders assessed the highest quality apps regarding usefulness (ie, does the app have the potential to help someone quit smoking) and usability (ie, it is easy to use).

## Methods

### App Selection

We identified all available smoking cessation apps in 2013 and randomly selected 100 for review. The top rated apps were then tested for usability and usefulness among 21 consenting smokers with psychotic disorders who were stable in mental health treatment. Although being videorecorded, each person used 2 randomly selected apps within a structured semiqualitative usability protocol, which lasted an average of 1 h. Videorecordings and text were analyzed to assess usability and usefulness of each app. State and University Institutional Review Boards approved and monitored the study.

**Figure 1:**
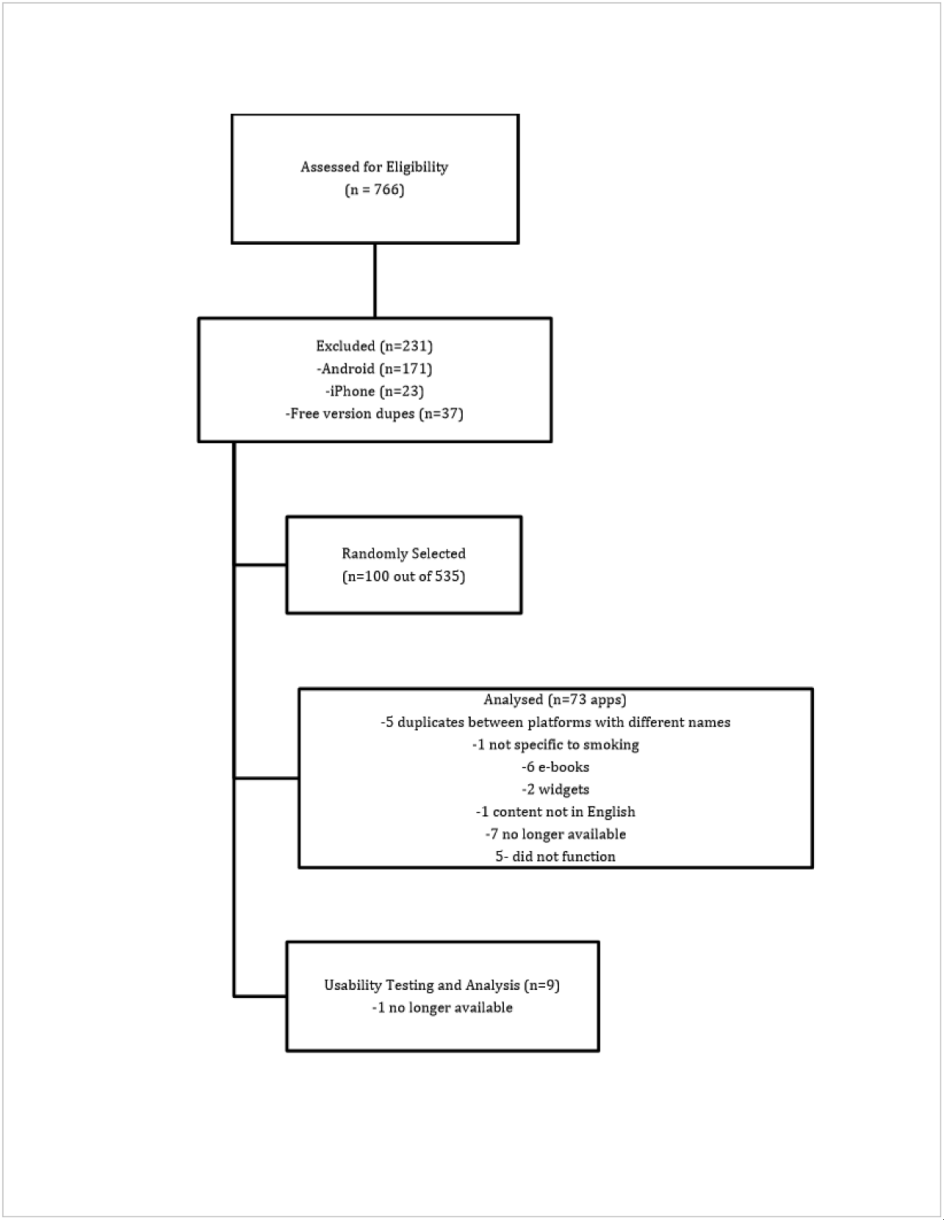
Mobile app screening flowchart.

First, using the search term “quit smoking” in both iPhone (iTunes App Store; sampled on July 15, 2013) and Android (Google-Play; sampled on July 11, 2013), we identified 479 app results from Android and 287 from iPhone. To be included in the review, the mobile app had to specifically address smoking cessation behaviors in English. On the basis of their brief written descriptions, we excluded apps based on the following findings: In the iTunes App search, 23 were excluded; of which, 8 were not in English, 8 were not related to smoking, 3 were books, 2 were about behavioral change but not specific to quitting smoking, and 2 were videos of burning cigarettes. In the Google Play search, 171 were excluded. Of the 171, 26 were marijuana related, 34 were widgets (eg, a component of an app, wallpaper, or other effects), 12 were videos of burning cigarettes, 17 were about acupuncture but not specific to smoking cessation, 42 were unrelated, 13 were generic for “bad habits,” 3 were books, 1 was specific to chew tobacco, 3 were videos, 12 were about hypnosis but not specific to smoking, 3 were not in English, 2 were mp4 files only, and 3 were stores selling tobacco using an app interface. A sample of 264 iPhone apps and 308 Android apps (572 total) were further assessed as follows (Figure 1).

Next, we match-merged the Android and iPhone files by name and publisher to discover overlapping mobile apps between platforms. We did not find any overlapping apps based on name of the app and publisher. Within platforms, many mobile apps were available in 2 versions (free version and upgraded version available for sale). We omitted the 37 free versions, maintaining the upgraded versions in the final group of 535 usable apps.

We randomly selected 100 apps for detailed review and rating. Upon detailed examination, we discovered 27 additional apps that were not eligible. These apps fit into the following three categories: (1) they were duplicates between platforms with different names (n=5); (2) they did not meet our study criteria— not specific to smoking (n=1), ebooks (n=6), in a different language — although title was in English (n=1), and widgets (n=2); or (3) they were not ratable because they were no longer available (n=7) or they did not function (n=5). The remaining 73 eligible apps are listed in Multimedia Appendix 1.

### Expert Assessments of Mobile Apps

We used the National Tobacco Cessation Collaborative rubric to define the type of the app (mobile app: calculators, calendars, hypnosis, rationing, and mixed types) [18]. Calculators were defined as a tool to compute how much money one spends on cigarettes per time period. Calendars were defined as a tool to track number of cigarettes smoked per day over time and to set quit dates. Hypnosis apps contained audio recordings of a person providing a hypnosis method. Rationing apps allowed users to set alerts to indicate when they can smoke each of their allotted cigarettes throughout the day. Mixed types perform two or more of the aforementioned functions. If the app did more than the functions specified within this classification system, the app was typed in the “other” category. We then performed a content analysis to identify subcategories within the “other” category—we found 9 additional types of apps: education, brain waves, motivation, games, virtual cigarette, virtual smoke, magic spells, graphic pictures, and social media (described in detail in “Results” section).

We used the 20-item Adherence to Practice Guidelines for Treating Tobacco Use and Dependence Index (ie, Adherence Index) [19]. Four responses were possible for each item (0=none; 1=minimal; 2=adequate; and 3=fully present). Two raters reviewed and rated each of the 73 apps (JF&PG). Interrater reliability was assessed with Cohen kappa and found to be excellent, .914 (SE .033), *P*<.001. We used an average of the two scores.

We then divided the Adherence Index into clinically relevant subindexes; each had adequate scores on Cronbach alpha. The first subindex, *Assessment of current use and attitudes* (Cronbach alpha=.61; 3 items) contained items such as, “Ask for tobacco use status.” The second subindex, *Enhancement of motivation* (Cronbach alpha=.87; 4 items) included items such as “Enhance motivation to quit with rewards.” The third subindex, *Advise every user to quit* (Cronbach alpha=.93; 4 items) contained items including, “Advise every user to quit with personalization.” The fourth subindex, *Assistance with quit plan* (Cronbach alpha=.89; 5 items) had items such as, “Assist with quit plan—used practical counseling.” The last subindex, *Referral to smoking cessation resources* (Cronbach alpha=.71; 4 items) included items such as “Refer to recommended treatment.”

### App Usability Assessment

As recommended by usability design experts [21], each app was assessed for usability by 3-5 users. In formative usability trials, use by a sample of 5 identified 80% of usability issues [22-24]. This study was designed to find out whether available cessation apps would be usable by a particular target population, not to identify all of the problems in the app, thus 3-5 was felt to be a parsimonious approach.

A total of 21 smokers with psychotic disorders provided informed consent. After brief assessment, researchers then guided participants through the usability assessment protocol. We provided a brief training on how to use a mobile phone. Using a basic weather app, participants were taught how to swipe, click, scroll, enter text, and get back to the homepage. When participants were able to complete these activities, they started the usability testing procedure on a sequence of two randomly selected smoking cessation apps. Following guidance from usability engineering [21], researchers instructed participants to use the mobile apps “as if they were trying to quit smoking” while “telling us what you think.” During this activity, participants were prompted to say their thoughts out loud [25]. The interviews were videorecorded with a focus on the participant’s hands and the screen of the mobile device. After using the apps, researchers used the Perceived Usefulness and Ease of Use scale [26] to assess participants’ perceptions of each app. It was found that 5-6 participants tested each app.

### Assessments—Participant Characteristics

During a structured interview, trained research assistants obtained participant demographics (age, level of education, race, ethnicity, and marital status), history of mobile phone use, and smoking activity. We assessed overall symptom level with the modified Colorado Symptom Index (CSI) [27], a 14-item questionnaire that measures psychiatric symptoms. The CSI has been found to be reliable and valid in people with mental illness and/or substance use disorder [28]. We obtained DSM-IV-TR psychiatric diagnosis from the medical record. We assessed level of nicotine dependence with the Fagerström [29] with scores from 0 to 10 (no to high dependence). After using each mobile app, participants responded to the Perceived Usefulness and Ease of Use subscales. This scale is an adapted 15-item questionnaire [26] and was used to gather reactions to and satisfaction with each app. The perceived usefulness subscale contains 11 items with a range from 11 to 55 with a higher score indicating better usefulness. The Ease of Use subscale contains 4 items with a range from 4 to 20 with a higher score indicating better ease of use (see Multimedia Appendix 2).

### Assessments—Usability and Usefulness

During the think-aloud protocol, researchers took field notes on usability issues (ie, difficulty with touch screen, typing, other) and usefulness (ie, opinions about if, how the app would, or could be used). Researchers also recorded observations on app use behavior through videorecording the participant’s hands, whereas they used each mobile app and wrote extensive field notes during and after each usability session. Researchers used the Flesch–Kincaid grade level scale within Microsoft Word to determine reading level for the text-heavy apps. This scale is standardized and uses word and sentence length to determine grade level [30].

### Analyses Plan

Basic descriptive statistics were used to describe both the mobile app sample and the participant user group (using SPSS v19). We used *t* tests to evaluate the differences between the types of app on the Clinical Guideline Index scores. We assessed the usability of the apps by evaluating usability themes within the videorecordings and field notes. We watched, transcribed, and coded the videorecorded data and field notes from each participant. We pulled quotes emblematic of users’ experience on each app. We developed themes that emerged from the data and found three categories of usability problems. Finally, we contrasted users’ qualitative descriptions of usefulness by deriving themes from quotes regarding apps that scored high on the usefulness scale and contrasting them to themes derived from apps that scored low.

## Results

### Expert Assessment of Mobile Apps

The 73 apps were categorized with the National Tobacco Cessation Collaborative rubric. Within the prespecified categories, nearly one-fifth of the apps were categorized as calculator (18%, 13/73), followed by hypnosis (12%, 9/73), mixed-type (combination of the descriptive categories; 10%, 7/73), rationing (5%, 4/73), and calendar (1%, 1/73). Over half of the apps fell into the “other” category (54%, 39/73).

Within the “other” category, almost half of the apps featured educational content (46%, 18/39) and a few had an additional interactive questions and. Many of the “other” category apps contained motivational content (20%, 8/39) with quotes or pictures aimed to help the smoker remember why they want to quit. Some apps claimed to change users’ “brain waves” with sounds (8%, 3/39), contained graphic pictures of smokers’ diseased organs (10%, 4/39), were virtual cigarettes or smoke (7.5%, 3/39), were games (3%, 1/39), were virtual “magic spells” (3%, 1/39), or contained elements of social support (8%, 3/39) either through social media or chat rooms.

Average scores of expert ratings on the Adherence to Clinical Practice Guidelines for Treating Tobacco Use and Dependence Index are shown in Table 1. Over two-thirds (about 69%) of the apps scored at or below 10 on a 60-point scale (Mean 11.47, SD 11.8, Range 0-51). Average guideline scores did not differ by platform (Apple vs Android). The average score was significantly higher for apps in the “other” category compared with the remainder of the types of apps (categories were collapsed due to small sample sizes; *t*_71_=2.21, *P*<.05). Within the “other” category, the education subtype of app scored higher than the other subtypes (*t*_37_=4.04, *P*<.001). The education apps also scored higher than other apps on the subindexes in every domain (Table 1).

**Table 1:** Mean scores on guidelines subscales in educational apps versus other.

Subtype	Total sample	Education	Other	*t* test	Degree of freedom	*P*
	mean (SD)	mean (SD)	mean (SD)			
n	73	18	55			
Assess	5.5 (2.6)	6.6 (2.3)	5.2 (2.7)	−2.14	33	.04
Enhance	2.7 (3.1)	5.5 (4.2)	1.8 (2)	−3.54	20	.002
Advise	2.2 (3.5)	5.1 (4.5)	1.3 (2.6)	−3.48	21	.002
Assist	1.7 (3.2)	5.2 (4.3)	0.6 (1.5)	−4.42	18	<.001
Refer	0.6 (1.8)	2.4 (2.9)	0.1 (0.5)	−3.35	17	.004
Total scale score^a^	11.5 (11.8)	23.2 (15.9)	7.7 (6.9)	−4.03	19	.001

^a^Unequal variances assumed.

A minority of apps contained content in 2 domains: (1) *assisting with a quit plan* and (2) *referring or connecting to recommended treatments*. In terms of *assisting with a quit plan*, about 19% (14/73) provided “practical counseling”—mostly by way of offering quit tips like, “Drink water when you have a craving for a cigarette.” Only 3 of them have provided instruction to perform a skill to help with quitting. For example, the app, Quit for Two, provides a picture of a baby blowing up a balloon in order to model deep breathing. Specific to *referring and/or connecting to recommended treatments*, 21% (15/73) of the apps mentioned smoking cessation medications and only 1% (1/73) recommended use of both medications and psychosocial treatment. It was found that 11% of the apps (8/73) referred participants to a Quitline.

The average cost per app was US $0.76 (SD US $1.21, Range: US $0 to US $4.99). Most apps were free (n=44, 60%). There was no relationship between the cost and Adherence Index scores (*r*=−.02, *P*=.88).

### Participant Characteristics

Most of the 21 participants were white (81%, 17/21) and male (81%, 17/21). Over three-quarters of the group (76%, 16/21) was diagnosed with a schizophrenia spectrum disorder; the remainder were diagnosed with bipolar disorder with psychotic features or psychosis not otherwise specified. The average amount of completed education was 12 years (SD 2) and most participants were unemployed (76%, 16/21). CSI scores indicated a moderate degree of mental illness symptoms (Mean 18, SD 12). Most participants were severely dependent on nicotine as measured by the Fagerström Nicotine Dependence scale (Mean 7, SD 2) and, on average, smoked 26 cigarettes per day (SD 9). Many had tried to quit in the past month (41%). Regarding use of phones and technology, most of the sample (81%, 17/21) owned a cell phone, 62% (13/21) owned a mobile phone, 43% (9/21) played electronic games, and 33% (7/21) of the group used social media.

### Usability

Through the think-aloud protocol, open-ended questions, and observations of participant’s use of the apps, 4 main themes emerged, of which 3 are related to design and 1 is related to content. First, one group of mobile apps were easy to use but were unappealing because they were text-heavy with minimal interactive features. A second group of apps were difficult to navigate due to main menus that featured abstract symbols, jargon, or one-word labels that the users did not understand. Third, many apps had subtle directions on how to use their interactive tools that users either failed to notice or did not understand. Finally, all but one of the apps were missing concrete directions on how to use quit smoking skills; although most suggested other things to do instead of smoking. We will expand on these themes and provide illustrative examples below.

#### Text-Heavy Design

Three of the apps consisted predominantly of text. These text-heavy apps seemed to be the easiest to use, but participants reported that they were boring and unengaging. For example, although expert reviewers rated Smoking Cessation Srior highly for breadth of smoking cessation information, participants had problems reading and understanding the text, which was at Flesch–Kincaid grade level (FCGL) 12. Two other apps consisted of a book-like format with easy to understand text (Quit Smoking Easily, FCGL=8.3 and You Can Quit Smoking, FCGL=6.4). Participants found them useful and easy to use, but boring, as exemplified by the comments “I’m getting tired of this app” and “I am bored.” Users indicated that they were unlikely to use this type of app.

#### Difficult Navigation

In contrast, many of the apps that held easily understandable, interactive content were difficult to navigate. The main menus of 4 apps (NCI QuitPal, San Francisco Stop Smoking, Quit for Two, Call it Quits) consisted of abstract symbols and one-word descriptions of each section (NCI QuitPal) or jargon-laden descriptors (Call it Quits). For example, Call it Quits called their homepage a “Dashboard,” which confused participants. When participants attempted to use these apps, they often did not know what the homepage buttons meant, requiring research staff assistance to continue. The abstract homepage titles and symbols were also poorly understood. Participants guessed as to section contents and were unable to find the information they sought.

#### Subtle Directions

Three apps featured subtle directions to use app features (Quit for Life, Smoke Free, and Call it Quits). These apps typically provided small buttons with symbols or one-word instructions as cues for how to use app features. Cue placement also impeded use; sometimes, the cues were off to one side of the page, which made them more obtuse. Participants experienced problems with subtle directions on how to enter their reasons for quitting, select quit tips, and choose motivations to quit. Many participants voiced frustration over these functions and said things like, “I can’t get this to work. How do I do this?” One participant stopped using the app, Call it Quits, because he could not get it to save the quit smoking tips he had selected, suggesting that subtle directions may be difficult to learn by this group.

#### Lack of Smoking Cessation Skills Training

Only one app provided content designed to help the user learn a cessation skill while using the app, whereas all the other apps simply provided brief instructions to do something different instead of smoking. The Quit for Two, Quit for You App illustrated deep breathing with a cartoon of a baby slowly inflating a balloon, providing in the moment instructions an effective skill to cope with craving.

**Table 2:** Participant ratings of app perceived usefulness and ease of use for top apps.

App Name		Perceived		Adherence			
		Usefulness	Ease of Use	Index	Text	Subtle	Difficult
	n	Mean (SD)	Mean (SD)	Score	Heavy	Directions	Navigation
NCI Quit Pal	5	42 (4.8)	18 (1.1)	51	0	0	x^a^
You Can Quit Smoking	5	39 (5.1)	16 (5)	49.5	x	0	0
San Francisco Stop Smoking	5	37 (7.8)	16 (4.4)	43	0	0	x
Quit Smoking Easily	5	36 (3.7)	16 (4.2)	40	x	0	0
Quit For You – Quit For Two	3	43 (5)	15 (1.7)	39	0	0	x
Quit For Life	5	38 (6.6)	15 (3.8)	36	0	x	0
Smoke Free – Stop Smoking Now	5	32 (7.8)	11 (5.9)	34	0	x	0
Smoking Cessation – SRIOR	6	31 (7.9)	14 (2.4)	31.5	x	0	0
Call It Quits	5	31 (11)	11 (4.6)	29.5	0	x	x

^a^x indicates the presence and 0 indicates the absence of usability issues.

### Perceived Usefulness and Ease of Use

Usefulness and ease of use scores are shown in Table 2. App usefulness ratings correlated with app quality (Adherence Index scores; *r*.34, *P*.01). Participants rated NCI Quit Pal and Quit for Two highly for usefulness. Participant comments provided examples on how they found the apps useful, including, “I would use Facebook to connect with friends and would personalize the settings to remind me what I’m saving for,” and “Use the tracking, savings goals, facts and tips for urges and quit lines (to quit smoking).” With the Quit for Two App, one person commented that it, “gives you tips that you can practice” and another said that it, “reminds you of your money saved and gives you good tips plus there are games to keep you busy.”

In contrast, the lowest rated apps on the usefulness scale were Call it Quits and Smoking Cessation Srior, which both scored 31. Participants stated, “You know what it (nicotine) does but that doesn’t help (with quitting),” “It’s like a book, you can only use the content once.” Call it Quits had more interactive tools, and participants commented that they would use the quit tips and reminders within this app, but most of them could not figure out how to do this because of the subtle instructional cues, which undermined the apps’ usefulness.

## Discussion

### Principal Findings

In this study of expert-rated quality and user-rated usefulness and usability, we identified multiple barriers indicating that currently available smoking cessation apps may be inaccessible or ineffective for most smokers with psychotic disorders. Although the top 9 apps scored moderately high on expert-rated quality, they performed poorly during user testing. We found 3 primary design flaws: text heaviness, subtle directions, and abstractions on the homepage.

A myriad of smoking cessation apps are available, leading to a high level of consumer choice, but we found several indicators likely to cause consumer confusion. First, we found that descriptions of 25% of the 100 randomly sampled apps were inaccurate. Second, we found that most apps scored low on content quality. Similar to Abroms’s results [18,19], the apps evaluated in this study performed best on the assessment of user smoking behaviors and poorly on all of the other subindexes of adherence to treatment guidelines. Much like Abrom’s findings, most apps did not inform users about smoking cessation medication or Quitlines (which are universally available in the United States), and strikingly, most apps did not provide adequate quit skills training. Since apps on the market do not have any indicator of whether they contain evidence-based content, consumers have no way to find and select the minority of apps with effective content. Concrete guidelines for app evaluation could ameliorate this situation [31].

Similar to previous research of website usability [7,9,10,32], we found that smokers with psychotic disorders had difficulty using apps. Although we found similarities with Rotondi’s work on usability of websites among people with schizophrenia [9], we also found differences. Rotondi [9] has suggested that scrolling is more usable than paging in this population, but users in this sample did not perform poorly with paging. Additionally, Rotondi has suggested that hyperlinks should be used. In this sample, most users did not understand hyperlinks. Also, apps with subtle directions scored lowest on the Adherence Index by the experts and were frustrating for users. Previous work on website usability [10,32] indicates that explicit instructions improve usability for people with psychotic disorders.

Several study design issues warrant further discussion. A small number of participants rated each app. The sample size of 3-5 users is supported by recommendations of usability design experts [21] and, in formative usability trials, a sample of 5 was found to identify 80% of the usability issues [22-24]. Our usability findings are supported by our quantitative data and other researchers’ findings [10], indicating that the sample utilized here provides a reasonable assessment of the apps. However, a larger sample would likely have found additional problems. Additionally, the scope of our usability study was limited to short-term use; the next steps in usability testing should include long-term use. Further, we did not evaluate efficacy. Efficacy testing in user populations with the highest rates of smoking is sorely needed.

### Conclusions

In summary, this study provides an updated evaluation of smoking cessation app quality, indicating ongoing poor quality of most apps and suggesting need for a system to inform consumers about whether apps contain content that is likely to be effective. This study also suggests that adults with psychotic disorders are unlikely to be able to use the highest quality apps. In order for apps to be effective for populations who have cognitive impairments, future app content should provide (1) motivational enhancement exercises and information, (2) recommendations about smoking cessation medications and other relevant support, and (3) information and instruction on how to cope with withdrawal and urges to smoke. App designs should utilize a balance of text and simple designs that facilitate ease of navigation and content comprehension. Smokers with schizophrenia may then obtain adequate, accurate, and useful information about their smoking and learn methods to quit.

## Appendix

Multimedia Appendix 1 Random sample of smoking cessation apps.

Multimedia Appendix 2 Perceived Usefulness and Ease of Use subscales.

## Abbreviations

CSI: Colorado Symptom Index
FEDM: Flat Explicit Design Model
