# Supplementary material for: Mobile Phone Apps for Smoking Cessation: Quality and Usability Among Smokers With Psychosis
Source: JMIR Hum Factors. 2017 Mar 3;4(1):e7. doi: 10.2196/humanfactors.5933 (PMC5357319; doi:10.2196/humanfactors.5933)
Supplement: Multimedia Appendix 1 [file humanfactors_v4i1e7_app1.pdf]

Appendix A

**Mobile Application Name**	**Publisher**
NCI QUIT PAL	NATIONAL CANCER INSTITUTE
SMOKING CESSATION - SRIOR	MAGNA HEALTH SOLUTIONS/MEDICAL
SAN FRANCISCO STOP SMOKING	UCSF
QUIT SMOKING EASILY	KOOLAPPZ/HEALTH & FITNESS
QUIT FOR YOU - QUIT FOR TWO	BCM PARTNERSHIP/HEALTH & FITNESS
QUIT FOR LIFE	ALERE WELLBEING, INC.
CIGA LOTTERY	MIGNON
SMOKE FREE - STOP SMOKING NOW	DAVID CRANE
YOU CAN QUIT SMOKING	KOOLAPPZ/LIFESTYLE
CALL IT QUITS	RIESTER
STOP SMOKING	CX WIFI MALAYSIA TEAM/HEALTH & FITNESS
HOW TO STOP SMOKING AIDS	VIJAY KUMAR/BUSINESS
QUIT SMOKING	CENTIRION MOBILE LTD./HEALTH & FITNESS
WECANSTOP SMOKING	DAVID AZOULAY
ATTA	SPARKNET INTERACTIVE
IQUITSMOKING!	IGNACIO BORRAJO
LETS QUIT	DAVE GROSMANN
QUIT SMOKING FAST REPORT	TWALLACEGPLAY/HEALTH & FITNESS
HOW TO STOP SMOKING NOW	NICHOLAS GABRIEL/HEALTH & FITNESS
STOP SMOKING ASSISTANT	ENOORDHUIZEN.NET/HEALTH & FITNESS
KWIT - QUIT SMOKING IS A GAME	NICOLAS LETT/HEALTH & FITNESS
QUIT SMOKING	INSPIRE IT SOLUTIONS, LLC/MEDICAL
QUIT SMOKING, STOP SMOKING HYPNOSIS	NEW LIFE MINISTRIES (US)
QUIT SMOKING WHITH SOLOE	FULL THRUST IGNITION/HEALTH & FITNESS
STOP SMOKING HYPNOSIS AUDIO	VISTA CONCEPTS. LLC/HEALTH & FITNESS
SINCE IQUIT	DUNK LTD
TWEET SMOKING	PLUSR INC.
SMOKER REDUCER QUIT SMOKING	NOCHINO DIGITAL/HEALTH & FITNESS
QUIT SMOKING MANAGER	VITO BELLINI
ONELESS	OLIVER NAUROTH
NON SMOKING ASSISTANT	HIGT/HEALTH & FITNESS
STOP SMOKING (THE BEST NO SMOKING APP TO TRACK	IOS DEVELOPER
THE PAINLESS STOP SMOKING CURE	JOHN TEMPLE GROUP PTY LTD.
QUITITUDE - STOP SMOKING	EIDOLON INDUSTRIES/HEALTH & FITNESS
IQUIT COUNTER	DOCSOFT APPS
CIGARETTE CONTROL & COUNTER	VOSTROP/HEALTH & FITNESS
QUIT SMOKING WITH ANDREW JOHNSON	MECHAEL SCHNEIDER
KICKIT- QUIT SMOKING	MINICORP
STOP SMOKING WITH SELF HYPNOSIS	HYPNOSIS AND MEDITATION FOR SUCCESS, LLC
ZOMBIE SMOKEOUT	THRUST INTERACTIVE

STOP SMOKING CALCULATOR	APPKINGDOM/FINANCE
CIGARETTE COUNTER	GEOLINX LLC/HEALTH & FITNESS
STOP SMOKING! NO SMOKING	ALEXEY KULESHOV/HEALTH & FITNESS
TYZEN THE HYPNOTIST	MEDL MOBILE
QUIT SMOKING TOMORROW	ROCKMOB SOFTWARE/HEALTH & FITNESS
QUIT SMOKING	JERE PARKER/HEALTH & FITNESS
THE JOY OF QUITTING SMOKING PRO	MUBIQUO
HOW TO QUIT SMOKING - DIABETES	PATIENT EDUCATION PROGRAMS LLC/MEDICAL
STOP SMOKING TALKING CIGARETTE	NBAPP/HEALTH & FITNESS
A BREATH OF FRESH AIR	A BREATH OF FRESH AIR
STOP SMOKING NOW	TAPMEDIA/MEDICAL
ANTISMOKING WARNING	FERNANDO GAIOFATO/HEALTH & FITNESS
HYPNOSIS HOW TO STOP SMOKING	HYPNOSIS SIMPLY/SPORTS
I QUIT SMOKING - GRAPH TRACKING	IKNOWLEDGESOLUTIONS
SMOKED BBOX	ALBERTO BARRERA JIMENEZ
SHOCKING SMOKING FACTS	STROIKA
GOTTA SMOKE?	PRIME73 INC.
IQUITSMOKING	EUROSONIC
STOP SMOKING.	SOCIAL SYNAPSE/HEALTH & FITNESS
QUIT WITH ME	DONOHARM APPS
COME ON!!	IHOMESOFT
AFFIRMATION BOOK - VICKI'S MAGIC	MAGIC SPELLS NOW
STOP SMOKING + STRESS HYPNOSIS	JBMARKETING/HEALTH & FITNESS
STOP SMOKING HYPNOSIS	ON BEAT LIMITED/HEALTH & FITNESS
STOP SMOKING. SUBLIMINAL	T3 APPS
HABITS AND HEALTH	NINHAO ONLINE CORPORATION
NO SMOKING DIARY	EONSOFT/LIFESTYLE
MY SMOKING LOG	CX WIFI MALAYSIA TEAM/LIFESTYLE
CIGARETTE SMOKE (FREE) -	WEED TIME/CASUAL
SMOKE A BOWL CIGARETTE	ANDROIDITY.COM/HEALTH & FITNESS
MOTIVATION FOR LIFE	MANERAN/LIFESTYLE
ADDICTION HELP BRAINWAVETRIAL	IMOBLIFE INC./HEALTH & FITNESS
BINAURAL BEATS: HABIT BREAKER	INNER NODE STUDIOS
STOP SMOKING NICOTINE WITHDRAW	5 STAR/HEALTH & FITNESS
