# Supplementary material for: Mobile Phone Apps for Smoking Cessation: Quality and Usability Among Smokers With Psychosis
Source: JMIR Hum Factors. 2017 Mar 3;4(1):e7. doi: 10.2196/humanfactors.5933 (PMC5357319; doi:10.2196/humanfactors.5933)
Supplement: Multimedia Appendix 2 [file humanfactors_v4i1e7_app2.pdf]

Appendix B

Thank you for using mobile phone applications for smoking cessation. We are interested in your thoughts and opinions about these applications. We are going to ask you some questions. There are no right or wrong answers. We just want to know what you think. First I am going to read some statements about using the cell phone app. Please say how much you agree or disagree with each statement. This card shows the response choices for each statement. You can say the number or the words.

1= totally disagree 2= disagree 3=neither agree nor disagree 4=agree 5 = totally agree

Z1. Using the app has helped me be aware of all of the methods to quit smoking Z1._____

Z2. Using the app helped improve my chances of quitting smoking Z2.______

Z3. Using the app helped me understand the effects of smoking on my body Z3.______

Z4. Using the app helped me understand the costs of smoking Z4.______

Z5. Using the app helped me be aware of the good things about smoking Z5.______

Z6. Using the app helped me be aware of the bad things about smoking Z6.______

Z7. Using the app will make it easier to quit smoking Z7.______

Z8. The application gave me too much information Z8.______

Z9. The app did not give me enough information Z9.______

Z10. The application not tell me what I wanted to know Z10.______

Z11. The app gave me all the information I needed Z11.______

Z12. I could easily understand the information the app presented Z12.______

Z13. I found it easy to get the app to do what I want it to do Z13.______

Z14. It was easy for me to become skillful at using the app Z14.______

Z15. I found the app hard to use Z15.______
